# A Case of Diseased Kidney, with the Post-mortem Appearances, Abridged from the Case-book of the Hospital of the Kentucky Penitentiary

**Published:** 1845-05

**Authors:** W. C. Sneed

**Affiliations:** Frankfort, Ky.


Art. III.—*A Case of Diseased Kidney, with the Post-mortem appearances*, abridged from the case-book of the Hospital of the Kentucky Penitentiary. By W. C. Sneed, M.D., of Frankfort, Ky.

Glass, ætat. 26, of small stature, pale complexion, light hair, engaged by the keepers as a wagoner, was taken very ill on the night of the first of October, 1844. He complained of violent pain in the right side, and had considerable irritability of the stomach. The day previous to his attack he had eaten very freely of apples and drank a large quantity of sweet cider. He attributed his attack to this cause, and the steward of the hospital treated him accordingly. He had an emetic administered and afterwards a cathartic which brought off, each way, large quantities of undigested apples and cider; some relief was obtained, but it was only temporary as the pain again returned with increased violence, and persisted with more obstinacy. Venesection was resorted to, and about 24 ounces of blood taken from the arm. This, with the use of antispasmodics and opiates, gave him a truce which lasted until the evening of the next day. On the return of the pain we were called to him, and after making thorough examination we had no hesitation in declaring the case one of colic, brought on by the too free indulgence in apples and cider. There was evident tympanitis in the right side which we supposed to be owing to collection of wind in the cæcum and ascending colon. Percussion gave the sound of tympanitis, which with the other symptoms and facts in the case led us to believe, without any hesitation, that our conjectures were well founded. We resorted to the use of opiates and tartar emetic, and in a few hours succeeded in subduing the pain. His pulse during the remission of pain would be full and hard, but on the recurrence of pain, it would become extremely small and rapid. When the pulse was full the skin would be hot, and *vice versa*. The irritability of the stomach ceased in a day or two, and did not recur during the remainder of his illness. The violent symptoms gradually disappeared, and in the course of a week he seemed to be in a fair way to recover. The side, however, continued to enlarge very gradually, and by the end of the second week the abdominal parietes had become elevated above a level with the ribs. The enlargement gradually extended towards the left side, and by the end of the third week had crossed the median line about one and a half inches. Percussion now gave a much flatter sound than it had heretofore. His bowels could be moved without difficulty, and his appetite had become very good. His stomach was perfectly quiet and performed its functions without difficulty.

It occurred to us about this time that our diagnosis was probably incorrect, and that the enlargement depended upon some other cause. We made some enquiries of the patient in regard to his previous history, and learned from him that when a boy he had received a kick from a horse on the right side, and that there had been considerable enlargement of the side ever since. This statement induced us to believe that the enlargement might depend upon disease of the kidney but we could not tell how it could be so rapid in its formation, and increase to such an immense size.

The enlargement continued to increase, and the patient to emaciate, until the thirty-fourth day after his attack, when he expired.

*Sectio-cadaveris*—24 hours after death. The body extremely emaciated; the muscles soft and flabby; a general petechial eruption over the surface. The chest presented nothing very remarkable. The heart was atrophied, pale and soft.

Abdomen.—On opening the cavity we were surprised at finding the viscera all crowded over into the left side. The liver, forced from its normal position, occupied the epigastric and left hypochondric region; it was somewhat atrophied but otherwise healthy.

Stomach about half its natural size, but healthy. The cæcum and ascending colon were on the left side of the vertebral column, and attached to the immense sack which proved to be the right kidney degenerated and filled with pus. This enormous sack was attached at its upper portion to the liver and diaphragm; on the left, to the mesentery, large and small intestines; posteriorly it was fastened down by adhesion to the spinal column; and in the same manner to the right side, and to the abdominal parietes as far over as the median line. At its lower end it was attached to the brim of the pelvis, and a portion of it occupied about one-third of that cavity. No vestige of urethra could be found. The left kidney hypertrophied and healthy.

The sack contained a few ounces over two gallons of pure bland pus. The pelvic portion of the kidney seemed to have undergone the greatest change. It was immensely enlarged, and must have contained nearly the whole amount of the pus. On the inner portion of the sack there were several bony deposits, some of them very firm and as large as a thumb nail. Seven orifices large enough to admit a finger passed from the body to the pelvic portion of the sack. The holes answered to the places of the papillæ and were not connected to each other. The parietes of the sack were not thicker than the human skin, and were filled with veins and arteries. A preparation was made of the specimen, and is now in the possession of Professor Cobb, of the Medical Institute of Louisville.

Frankfort, April 1845.

